# p53 Amino-Terminus Region (1–125) Stabilizes and Restores Heat Denatured p53 Wild Phenotype

**DOI:** 10.1371/journal.pone.0007159

**Published:** 2009-10-22

**Authors:** Anuj Kumar Sharma, Amjad Ali, Rajan Gogna, Amir Kumar Singh, Uttam Pati

**Affiliations:** Transcription and Human Biology Lab, School of Biotechnology, Jawaharlal Nehru University, New Delhi, India; University of Arkansas for Medical Sciences, United States of America

## Abstract

**Background:**

The intrinsically disordered N-ter domain (NTD) of p53 encompasses approximately hundred amino acids that contain a transactivation domain (1–73) and a proline-rich domain (64–92) and is responsible for transactivation function and apoptosis. It also possesses an auto-inhibitory function as its removal results in remarkable reduction in dissociation of p53 from DNA.

**Principal Findings/Methodology:**

In this report, we have discovered that p53-NTD spanning amino acid residues 1–125 (NTD125) interacted with WT p53 and stabilized its wild type conformation under physiological and elevated temperatures, both *in vitro* and in cellular systems. NTD125 prevented irreversible thermal aggregation of heat denatured p53, enhanced p21-5′-DBS binding and further restored DBS binding activity of heat-denatured p53, *in vitro*, in a dose-dependent manner. *In vivo* ELISA and immunoprecipitation analysis of NTD125-transfected cells revealed that NTD125 shifted equilibrium from p53 mutant to wild type under heat stress conditions. Further, NTD125 initiated nuclear translocation of cytoplasmic p53 in transcriptionally active state in order to activate p53 downstream genes such as p21, Bax, PUMA, Noxa and SUMO.

**Conclusion/Significance:**

Here, we showed that a novel chaperone-like activity resides in p53-N-ter region. This study might have significance in understanding the role of p53-NTD in p53 stabilization, conformational activation and apoptosis under heat-stress conditions.

## Introduction

The tumor suppressor p53 protein is a transactivator that contains an independent regulatory N-ter domain (NTD) of approximately hundred amino acid (aa) residues affecting its activity and thermostability [1], [2]. There is substantial lack of structural and biophysical information on N-ter domain; in particular, this domain appears to be completely disordered with the typical features of the natively unfolded protein [3]. p53-NTD contains a transactivation domain (TAD, spanning aa residues 1–73) that alters transcription of genes controlling cell cycle arrest, proliferation and apoptosis [4], [5], and a proline rich domain (PRD, spanning aa residues 63–92) that plays role in drug induced, p53 mediated apoptosis [6] and influenced the ability of central domain to bind to DNA [7]. It was proposed that TAD is composed of rapidly equilibrating conformers, one quasi-globular and the other relatively open, this intrinsically disordered domain with a tendency for helical structure in the TAD1 (aa residues 18–25) becomes helical on binding to MDM2 [8]. p53-NTD also possesses an auto-inhibitory function that controls the dissociation of p53 from DNA binding site (DBS) as the removal of 96 aa residues exhibits a remarkable reduction in dissociation from DNA [9] and the deletion of 40 aa residues changes the stability of p53 at 4°C [2]. A 20 aa region spanning 101–120 aa residues was shown responsible for thermostable phenotype of human p53, that could partially protect the PAb1620^+^ conformation of tumor-derived p53 mutant from thermal unfolding [10]. An additional negative regulatory region of p53 sequence-specific DNA binding was identified in proline rich region spanning aa residues 80–93, furthermore, synthetic peptides from this region (aa 80–93) are able to activate p53 DNA binding activity *in vitro* [11]. A peptide derived from p53-N-ter region (aa residues 105–126) inhibited p53 DNA binding and interfered with p53 DNA binding that was activated by PAb421 antibodies [12]. The activation of DNA binding function of p53 is not synonymous with protection of thermal denaturation; however, these functions may be used in cells to control the physiological activities [13]. Further, NTD is essential for the activity of WT p53 in apoptosis [14] including p53-mediated neuronal cell death [15]. On the contrary, a p53 natural isoform, that was deleted of 40 aa in NTD domain (ΔNp53), was identified in mammalian cells lines; and in normal cells, it was shown to be tumoriogenic and deficient in transactivation of MDM2 and p21 genes [16]. This isoform didn't form complex with MDM2 and failed to accumulate in response to DNA damage.

Heat denaturation of WT p53 and a majority of amino acid substitutions in p53 that occur in tumor destabilize the native DNA-binding conformation of core domain [17]. As NTD (1–73) is responsible for transactivation, it is interesting that through this portion of the molecule the sequence specific DNA binding of p53 must be stabilized. *In vivo*, this could result in reduced dissociation and increased association of p53 under conditions requiring the activation of specific genes for specific function. The cryoelectron microscopy study of full length p53 protein, that claimed to represent the *in vivo* nature of p53 oligomerization, reveals that aa 1–100 of N-ter of one monomer appears to abut the last aa 323–393 of C-ter of the partner in the dimer forming N/C nodes [18]. As intrinsically disordered segments of chaperones such as α-synuclein and casein become ordered due to reciprocal entropy transfer by contacting mis-folded part of the substrate [19]–[21], we explored whether disordered p53-NTD might possess chaperone-like activity and have any role in stabilizing DBS-binding conformation of WT p53.

In this report, we have discovered a novel function of NTD125 that binds to WT p53, stabilizes and restores its wild type conformation *in vitro* both at physiological and elevated temperatures. In cells, NTD125 stabilized and activated cytoplasmic p53 in initiating its nuclear translocation that led to activation of p53 downstream genes. Exogenously supplied NTD125 thus possessed a chaperone-like activity that activated p53 and could be of significance in restoring p53 mutant phenotype in cells under stress.

## Results

### NTD125 exhibited thermostability and physically interacted with p53

**Figure 1:**
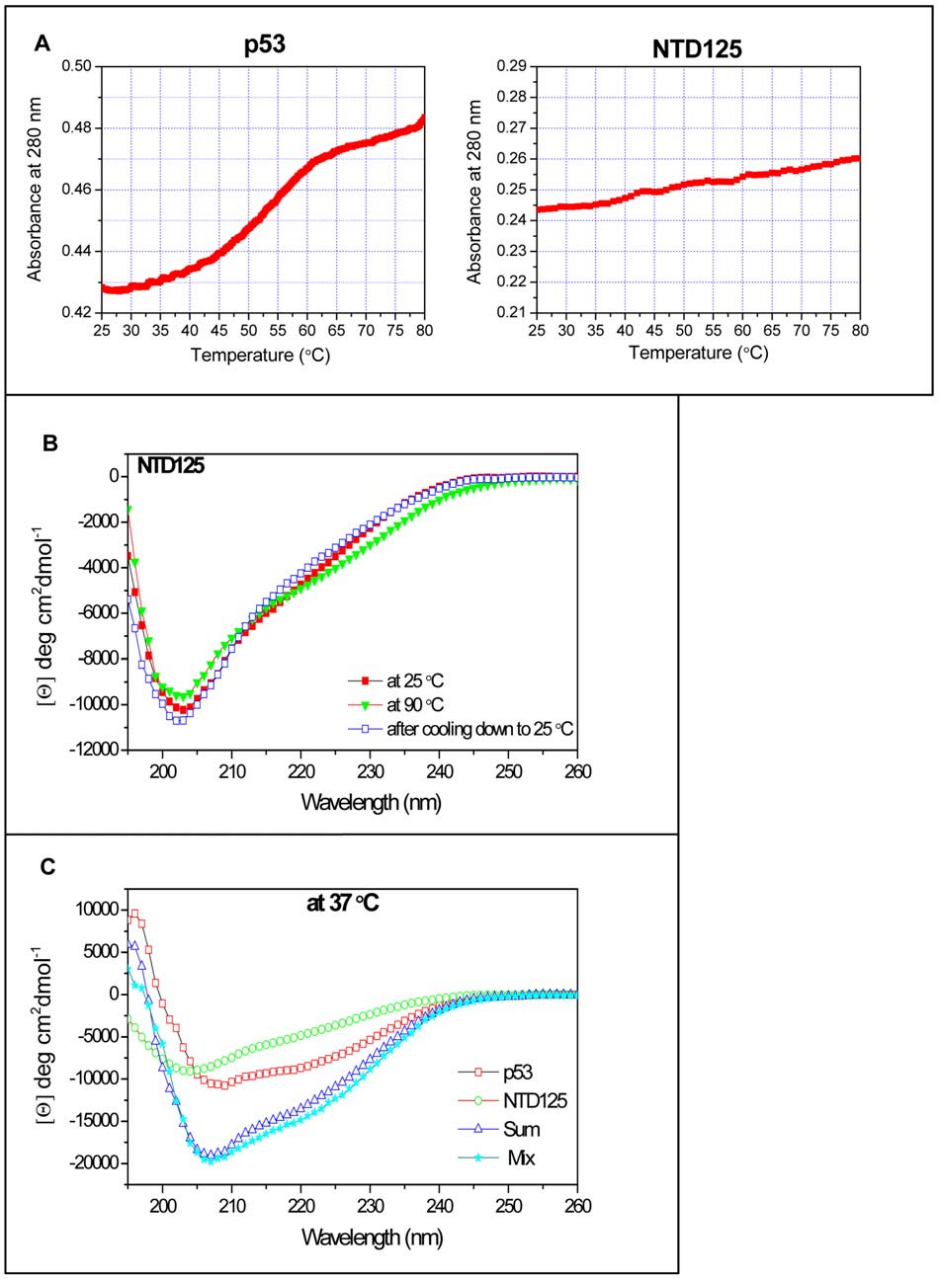
NTD125 is highly thermostable. A. UV spectra of p53 and NTD125. p53 starts melting at ∼35°C whereas NTD125 shows no distinct unfolding. B. Far-UV-spectra of NTD125 at 25°C (▪), 90°C (▴) and after cooling down to 25°C (□), shows that NTD125 was a less structured peptide and its secondary structure was reversible after cooling it down to 25°C. C. CD spectra analysis of p53 (□), NTD125 (○), theoretical sum of both (Δ) and mixture of both (░). The spectra of mixture were significantly different from the theoretical sum of individual spectra of NTD125 and p53 at 37°C; thus suggesting physical interaction between the proteins.

**Figure 2:**
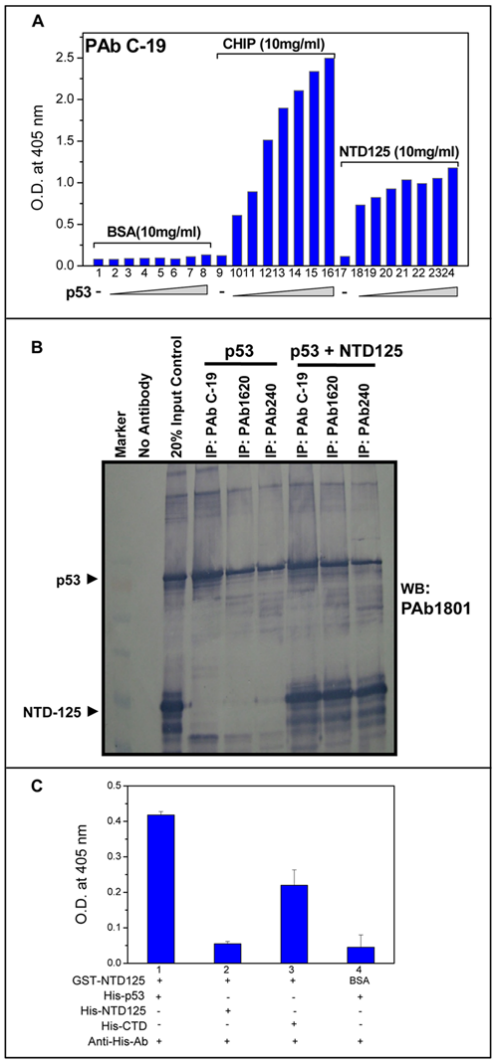
NTD125 interacts with p53. A. ELISA showing interaction between NTD125 and p53. 50 µl of 10 µg/ml protein (BSA, lanes 1–8; CHIP, lanes 9–16; NTD125, lanes 17–24) was coated on to the wells and then p53 was added in increasing concentration, ELISA was developed using anti-p53 specific antibodies PAb C-19. CHIP and BSA were used as positive and negative controls respectively. B. Co-immunoprecipitation assay showing interaction of NTD125 with different conformations of p53. C. ELISA showing interaction of GST-NTD125 with His-p53 and His-CTD (lanes 1 & 3), whereas GST-NTD125 is unable to interact with His-NTD125. BSA is used as negative control.

Highly purified recombinant p53 and NTD125 proteins were utilized for studying their thermostability and interaction. The thermal melting curve, recorded at 280 nm in a UV-visible spectrophotometer, showed that p53 started to melt at ∼35°C whereas NTD125 did not exhibit distinct melting pattern of folded proteins (Fig. 1a). Far-UV-CD spectra of NTD125 also showed that it is natively unfolded and secondary structure analysis predicted that it predominantly contained β-sheets (61.3%) and random coil (36.9%) at 25°C (Fig. 1b). Although NTD125 possessed some residual secondary structure as its conformational change at higher temperature (90°C) was reversible on cooling it down to 25°C (Fig. 1b). These results confirmed the thermostable nature of NTD125. Earlier studies with amino-terminus domain containing 1-99 aa residues also showed NTD as natively unstructured at physiological conditions and thermostable [3], [22]. Far-UV-CD spectra analysis (range 260–195 nm) was further utilized to check interaction between NTD125 and p53. The spectrum of mixture of both proteins was significantly different from the theoretical sum of individual spectra of NTD125 and p53 (Fig. 1c). It is widely reported in literature that if two proteins do not interact with each other, no structural change would result so the theoretical and experimental spectra would be identical. However, if the two proteins do interact substantially, conformational change in their structure would be detected and in this case, theoretical and experimental spectra would be substantially different. As it was shown in Fig. 1c, when p53 and NTD125 were combined at different temperatures, they interacted to produce evidence of a structural change. The same figure also presented individual spectra of p53 and NTD125. As significant structural changes had occurred due to probable interaction between these two peptides, we noticed the difference between spectra of mixtures and the theoretical sum of individual spectra. This observation led us to conclude that there was physical interaction between NTD125 and this interaction might further have stabilized p53 at higher temperatures (Fig. 1c, i–iv). Further, the interaction between p53 and NTD125 was studied by enzyme linked immunosorbent assay (ELISA). 0.5 µg of either BSA, CHIP or NTD125 protein was plated per well onto which p53 (in increasing concentration) was added, followed by detection with anti-p53 antibody (PAb C-19). NTD125 showed interaction with p53 in ELISA too (lanes 18–25). BSA (lanes 2–8) and CHIP (lane 10–16) were taken as negative and positive controls respectively (Fig. 2a). We then analyzed this interaction by immunoprecipitation (IPP) assay at room temperature using various p53 antibodies, PAb C-19 (specific for p53-C terminus), PAb1620 (specific for wild type p53) and PAb240 (specific for denatured/mutant p53); NTD125 was shown to bind to both p53 wild and mutant conformation with equal intensity (Fig. 2b). Again ELISA showed that NTD125 interacts with p53 and with C-ter domain (CTD) of p53 revealing that NTD125 interacts with p53 in C-terminus region (Fig. 2c). The interaction between NTD (1–186) and CTD (187–393) in cells was shown [18], and a low energy complex between CTD (361–382) and PRD (80–93) was predicted earlier [23]. The thermostable nature of NTD125 and its interaction with p53 led us to analyze whether it could stabilize p53 wild type conformation and DNA binding activity at elevated temperatures.

### In vitro stabilization of p53 wild type conformation at higher temperature by NTD125

**Figure 3:**
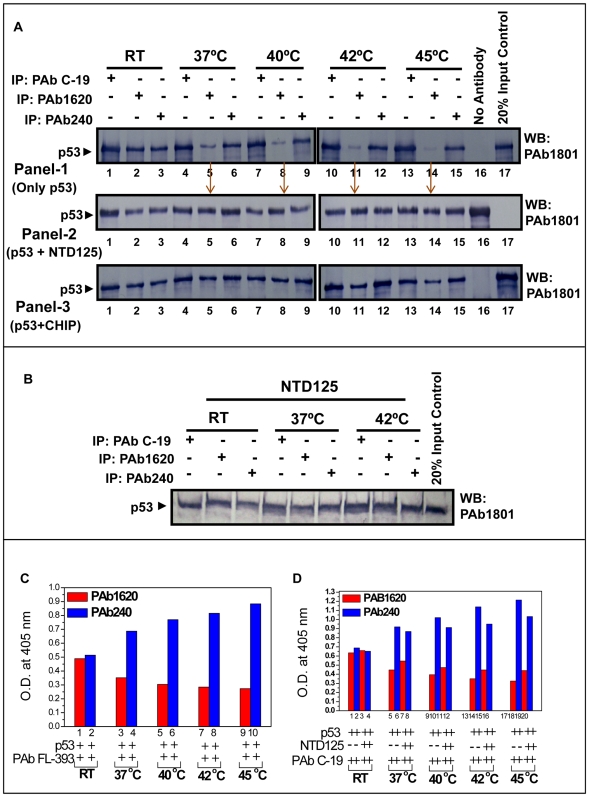
NTD125 stabilizes WT p53 *in vitro*. A. Loss of p53 wild type conformation (PAb1620) at higher temperature. Recombinant p53 was heated and conformational changes monitored with PAb1620 and PAb240 by IPP at (37–45°C); gradual loss of wild type conformation was observed (Panel-1, lanes 2,5,8,11,14 & 16), mutant conformation was stable at (RT-42°C) (Panel-1, lanes 3, 6, 9, 12) and decreased at 45°C (Panel-1, lane 15). In presence of NTD125 wild conformation (37–45°C) was protected (Panel-2, lanes 5, 8, 11, 14). Addition of recombinant CHIP (known chaperone of p53) also protected wild conformation of p53 (Panel-3, lanes 5, 8, 11, 14) at different temperatures (37–45°C) B. Further, addition of NTD125 post incubating p53 at various temperature, restores wild conformation (lanes 5 and 8) and C. ELISA showing rise in mutant conformation (lanes 4, 6, 8, 10) at increasing temperature and D. protection of wild conformation in the presence of NTD125 (lanes 7, 11, 15, 19).

We monitored the transition of wild type p53 conformation in to the mutant phenotype at elevated temperatures utilizing p53 conformation specific antibodies (PAb1620 and PAb240) by IPP and ELISA. The recombinant p53 preparation contained both wild and mutant phenotype at ∼1∶1 ratio; shown by IPP with conformation-specific antibodies PAb1620 (wild specific) and PAb240 (mutant specific) at RT (Fig. 3a, panel 1, lanes 2 & 3). The recombinant preparation was separately heated at 37°C, 40°C, 42°C and 45°C and the heated mixture was immunoprecipitated either with PAb1620, PAb240 or PAb C-19 antibodies. When p53 was heated sequentially from 37°C to 45°C, the PAb1620 form was lost (Fig. 3a, panel-1, lanes 2, 5, 8, 11, & 14). The loss of wild type (PAb1620) form was progressive with the rise in temperature; the loss of wild type conformation at 37°C and 40°C was about 80% whereas there was a total loss of this conformation at 45°C within 1 hour (Fig. 3a). When NTD125 (1∶5 molar ratio) was added prior to denaturation, there was no loss of wild type (PAb1620) (Fig. 3a, panel-2, lanes 2, 5, 8, 11, & 14, see arrow). In the presence of CHIP (1∶2 molar ratio), a known chaperone of p53, the wild type (PAb1620) form was not lost (Fig. 3a, panel-3). This suggests that NTD125 showed chaperone-like function in stabilizing wild type conformation (PAb1620). In a parallel experiment, p53 was first heated to denaturation for 1 h at 37°C followed by addition of NTD125 (Fig. 3b). In a similar manner, no loss of PAb1620 form was noticed thus confirming the chaperone-like activity of NTD125. This observation was further confirmed by sandwich ELISA in which conformational antibodies PAb1620 (wild type specific) and PAb240 (mutant specific) were plated, onto which heat-denatured p53 (incubated with or without NTD125) was added. After washing the unbound protein, bound wild and mutant phenotypes were probed with either FL-393 (polyclonal antibodies against full length p53) or PAb C-19 (p53 C-ter specific antibodies); PAb C-19 was preferred when NTD125 was added into the mixture. Similar results were obtained by ELISA experiment (Fig. 3c & d) in which addition of NTD125 resulted in an increase in wild type form (PAb1620) (Fig. 3d, compare lanes 5, 7; 9, 11; 13, 15; 17, 19) and decrease in mutant form (PAb240). The result was more prominent in IPP experiment (Fig. 3a, b) than ELISA. Thus, NTD125 shifted the equilibrium from the mutant to wild type and behaved as a chaperone-like peptide.

### NTD125 prevented irreversible thermal aggregation of p53

**Figure 4:**
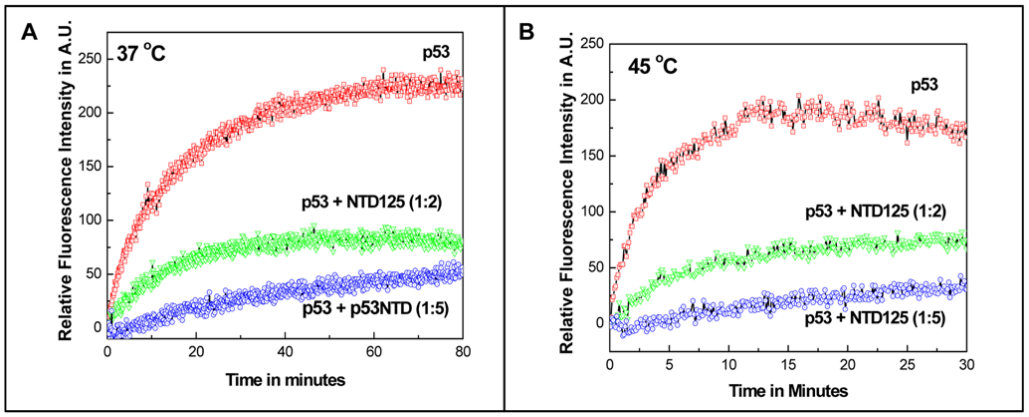
Prevention of irreversible p53 thermal aggregation by NTD125. A. & B. Time dependent fluorescence studies at 340 nm show that at 37°C and 45°C, NTD125 (at 1∶2 and 1∶5 molar ratio) prevented irreversible p53 thermal aggregation.

HSP90 [24], CHIP [25] were earlier shown to suppress p53 aggregation and catalyze disaggregation at elevated temperatures. As NTD125 was shown to bind to p53 and stabilized the wild conformation at elevated temperatures, we asked whether NTD125 could prevent aggregation of thermally denatured p53. p53 alone and with NTD125 (1∶2 and 1∶5 molar ratio) were incubated at 37°C and 45°C and the thermal aggregation kinetics was recorded by measuring light scattering in a fluorescence spectrophotometer. As WT p53 aggregated and reached a plateau after 40 minute at 37°C and after 10 minute at 45°C, addition of NTD125 in two molar excess prevented aggregation by approximately 75% both at 37°C and 45°C (Fig. 4a) whereas addition of five molar excess of NTD125 suppressed aggregation completely (Fig. 4b), thus suggesting that NTD125 stabilized p53 in shifting the equilibrium from the mutant to the wild type.

### NTD125protected and restored DNA binding of heat-denatured p53

**Figure 5:**
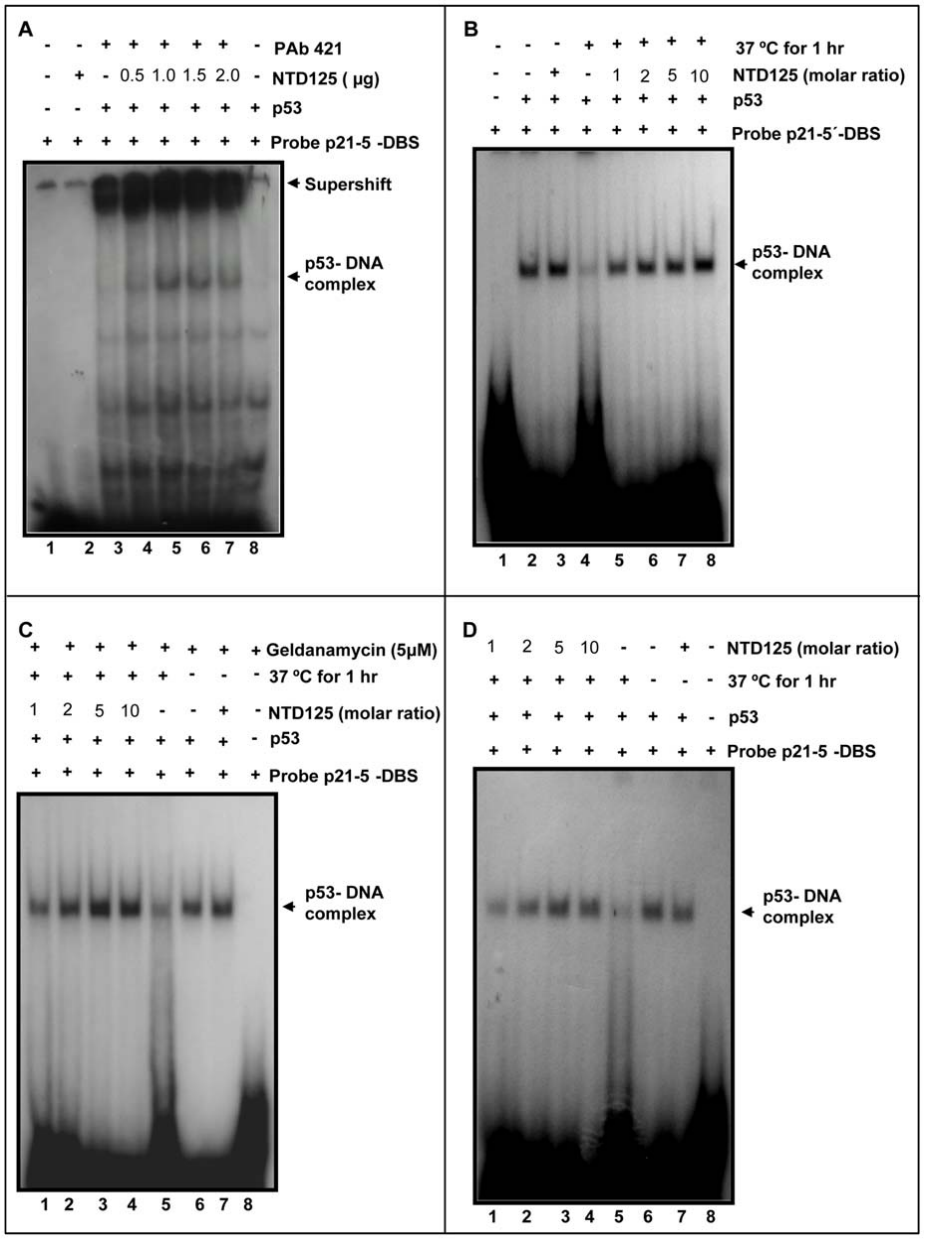
NTD125 stabilizes and restores DNA binding activity of heat denatured WT p53. A. EMSA showing role of NTD125 upon p53-DBS binding. 100 ng of p53 was incubated with 3 ng P^32^ labeled p21-5′-DBS with (lanes 4-7) or without (lane 3) PAb421. Addition of NTD125 (0.5- 2.0 µg) resulted in enhanced DBS binding (lanes 4–7). B. p53 DBS binding (lanes 2 & 3); loss of DBS binding after heating p53 at 37°C for 1 hour (lane 4); Stabilization of DNA binding occurred after addition of NTD125 prior to heating (different molar ratios, lanes 5–8). C. in presence of Geldanamycin (5 µM), a HSP90 inhibitor, the loss of DNA binding after heating p53 at 37°C (1 hour) (lane 4) and stabilization of DBS binding after adding NTD125 in increasing concentration (lanes 5–8). D. Restoration of DNA binding occurred after adding NTD125 (different molar ratios, lanes 1–4) post p53 denaturation step.

**Figure 6:**
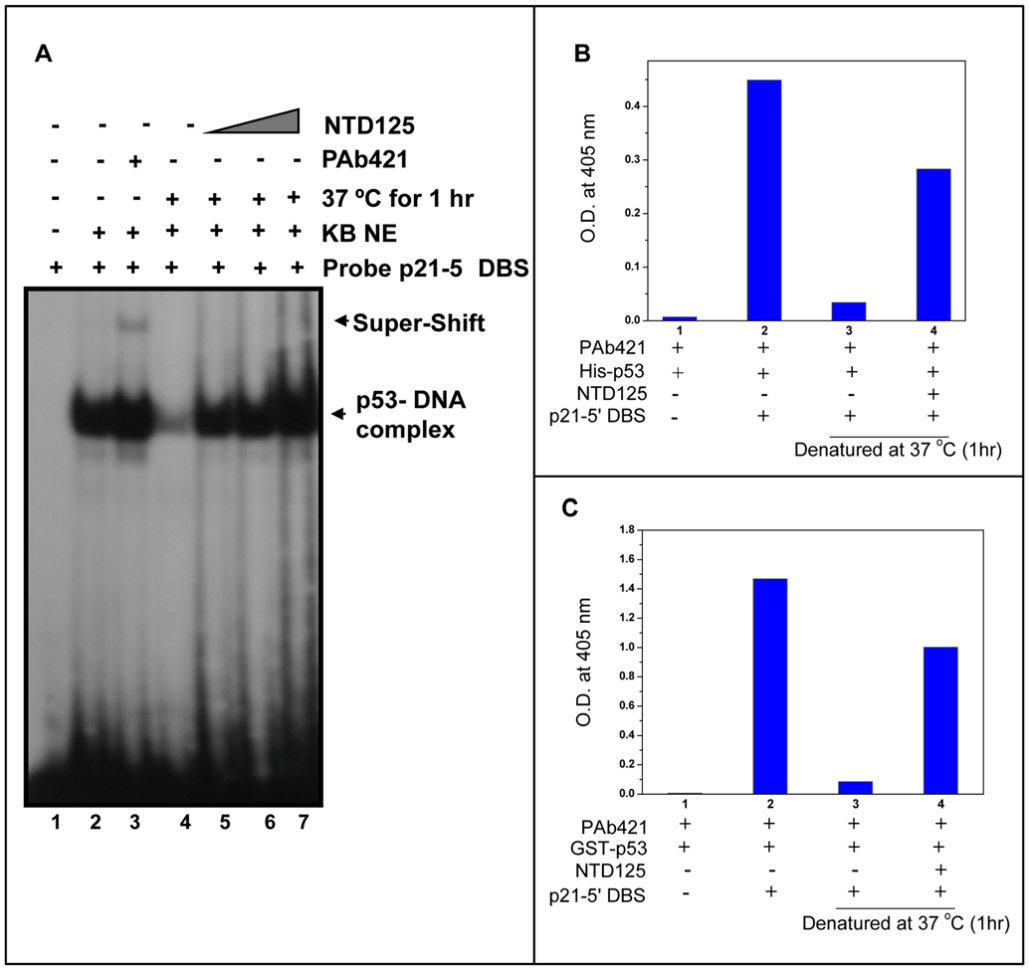
NTD125 stabilizes DNA binding activity of heat denatured native and recombinant p53. A. Lost activity of native p53 (lane 4) was also restored after adding NTD125 (lanes 5, 6 & 7) while using KB-NE as a source of WT p53. B. ELISA showing His-p53-DBS binding (lane 2), loss of DBS binding after heating p53 at 37°C (lane 3) and stabilization of DBS binding (lane 4). c. ELISA showing GST-p53-DBS binding (lane 2), loss of DBS binding of p53 that was heated at 37°C (lane 3) and stabilization (lane 4). PAb421 coated wells incubated with either p53 or heat-denatured p53 in presence or absence of NTD125 followed by addition of biotin-labeled p21-5′-DBS, color development was done with alkaline phosphatase conjugated Avidin.

In order to check the role of NTD125 upon DNA binding activity of p53, electrophoretic mobility shift assay (EMSA) was employed with or without NTD125 (in an increasing concentration), both under normal and denaturing conditions. p21-5′-DBS was radio-labeled and was mixed with recombinant p53 and DNA competitor for EMSA analysis either in the presence or absence of PAb421 antibodies (for super shift) and/or purified recombinant NTD125. An enhancement of DBS binding was observed after addition of NTD125 (0.5, 1.0, 1.5, and 2.0 µg) in the reaction mixture (Fig. 5a, lanes 4, 5, 6 & 7). After heating p53 at 37°C for 1 hour, no binding was observed (Fig. 5b, lane 4) as most of the p53 was in mutant conformation, lacking DNA binding activity. Addition of NTD125 prior to denaturation step (1∶1 to 1∶10 molar ratio) resulted in complete restoration of DBS binding at 10 molar excess (Fig. 5b, lanes 5–8). HSP90 and CHIP were earlier shown to chaperone WT p53 [24], [25], the EMSA was repeated in the presence of HSP90 inhibitor geldanamycin and after utilizing bacterially expressed p53 that was passed through anti-CHIP and anti-HSP90 antibodies column (to terminate the role of any CHIP and HSP90 like homolog from bacterial system); similar results were obtained (Fig. 5c). In a parallel experiment, p53 was first denatured followed by the addition of NTD125 (Fig. 5d). Identical results were obtained thus suggesting that NTD125 restored p53 wild form and might have chaperone-like activity. In addition, we have utilized KB (p53^+/+^) nuclear extract (NE) to check the effect of NTD125 on native WT p53. NE was first heated to denaturation in order to lose DBS binding and addition of NTD125 post denaturation resulted in restoration of DBS binding (Fig. 6a). Further, DNA-protein ELISA was utilized in order to study the interaction between biotinylated p21-5′-DBS and p53 in which either His-p53 or GST-p53 was used. Strong p53-DBS binding was detected with GST-p53 in comparison to His-p53 and both denatured GST-p53 and His-p53 failed to bind to DBS. Incubation of p53 with recombinant NTD125 prior to denaturation step resulted in protection of DBS binding by ∼60% (Fig. 6b & c), thus supporting the EMSA data described above. These results thus confirmed that exogenous NTD125 stabilized p53 native conformation and facilitated p21-5′-DBS binding. Although a direct physical association through IPP was observed between NTD125 and p53 wild as well as mutant phenotype; the binding (super shift due to NTD125) was not detectable in EMSA in the presence of DBS suggesting that NTD125 might bind to WT p53 transiently in order to modulate its conformation prior to DNA interaction.

### Stabilization of p53 wild phenotype in cytoplasm and nuclear translocation of activated WT p53 by NTD125

**Figure 7:**
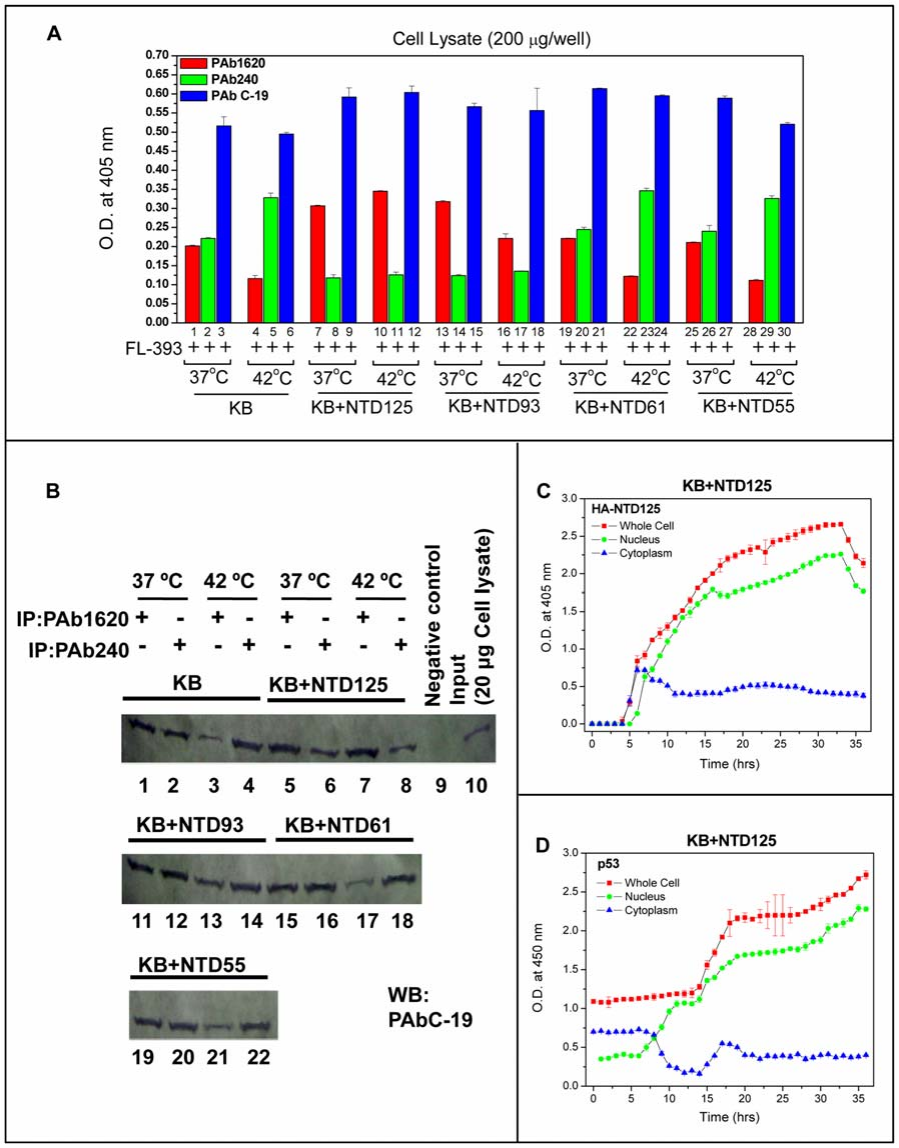
NTD125 activates and translocates cytoplasmic p53 in to the nucleus. A. *In vivo* ELISA showing NTD125 protecting p53 wild type conformation at physiological and elevated temperature. NTD constructs (NTD125, NTD93, NTD61 and NTD55) were transfected into KB cells and WCE (200 µg) was analyzed for estimating total cellular p53 (PAb C-19), p53 wild type (PAb1620) and mutant type (PAb240) conformation at various temperatures. Equal amount of wild and mutant form at 37°C (lanes 1, 2) and rise in mutant form at 42°C (lane 5) were observed whereas the presence of NTD125 and NTD93 decreased mutant form both at 37°C and 42°C (lanes 8, 11) and increased wild form (lanes 7, 10, 13); NTD61 and NTD55 failed to show chaperone-like function. B. Immunoprecipitation utilizing PAb1620 and PAb240 confirmed the above finding. NTD125 protected the wild form (PAb1620) when compared with KB control (compare lanes 3 & 7) at 42°C, NTD93 partially protected the wild form (compare lanes 1 & 11; 3 & 13); NTD 61 and NTD55 failed to protect the wild form at 42°C (compare lanes 3, 17 & 21). C. Time course ELISA showing p53 and NTD125 level post NTD125 transfection. PAb421 and HA-antibodies were used to estimate cellular (□), cytoplasmic (Δ) and nuclear protein (○) level at every hour post NTD125 transfection; NTD125 enter nucleus at ∼5^th^ hour after transfection; the estimated amount of both nuclear and cellular NTD125 was approximately equal (left panel). D. In a synchronized manner most of the cytoplasmic p53 enters nucleus at ∼6^th^ hour post NTD125 transfection and the total cellular p53 was equal to the total cytoplasmic p53.

The restoration of p53 wild type conformation by NTD125 in EMSA led us to ask whether p53 could be stabilized by NTD125 in cells. We found that in KB cells, over-expression of HA-NTD brought a rise in p53 level and there was cellular interaction between NTD125 and endogenous p53 (data not shown). A negatively charged peptide based chaperone strategy to rescue p53 mutant conformation was reported to raise and stabilize p53 level [26] although no reasonable mechanism was proposed. We discovered that, in KB cells, exogenous NTD125 stabilized p53 wild conformation and reduced the mutant phenotype both under physiological and elevated temperature (Fig. 7a & b). In order to identify the minimal region that was responsible for the observed chaperone-like function, we further expressed HA-tagged NTD125 and its deletion constructs (NTD93, NTD61, and NTD55) in KB cells and utilized the whole cell extract (WCE) for *in vivo* ELISA and IPP utilizing conformation specific antibodies (PAb1620/PAb240). *In vivo* ELISA, at 37°C, showed that the ratio of p53 wild type (PAb1620) and mutant (PAb240) was ∼1∶1 (Fig. 7a, lanes 1 & 2). At 42°C, the mutant was higher than wild type (Fig. 7a, lanes 4 & 5). In the cells that were tranfected with NTD125, the trend was reversed. The wild type was higher than the mutant form both at 37°C and 42°C (Fig. 7a, lanes 7, 8 and 10, 11). Similar results were obtained by IPP experiment both at 37°C and 42°C. At 37°C, the protein bands of wild type and mutant were of equal width (Fig. 7b, lanes 1, 2) whereas the mutant was higher than wild type 42°C (Fig. 7b, lanes 3, 4). In NTD125-transfected cells, wild type p53 was at higher ratio (Fig. 7b, lanes 5, 7) than the mutant (Fig. 7b, lanes 6, 8). In ELISA, NTD93 showed partial stabilization of wild type (Fig. 7a, lanes 13, 14; Fig. 7b, lanes 11, 12) at 37°C. However, NTD61 and NTD55 failed to show any protection. The above experiment thus established the chaperone-like stabilizing activity of p53-NTD that includes both the transactivation and proline-rich domain.

**Figure 8:**
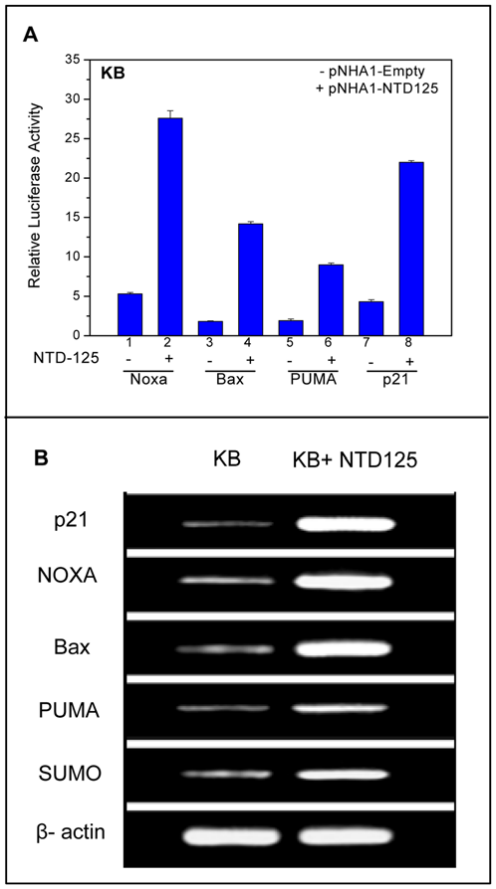
NTD125 mediated activation of p53 downstream genes. A. Luciferase assay showing NTD125-mediated activation of Noxa, Bax, PUMA and p21 promoters. The promoter-luciferase constructs were transfected with or without NTD125 constructs and luciferase activity was monitored. Approximate activation of Noxa, Bax, PUMA and p21 promoter was 5.0, 7.5, 4.5, and 5.0 fold respectively. B. Reverse-transcriptase (RT) PCR showing higher expression of p21, NOXA, Bax, PUMA and SUMO genes in NTD125 expressing cells in comparison to only KB cells. Experiment was performed 24 hours post NTD125 transfection.

Further, a time course analysis of NTD125 and p53 protein level in nuclear and cytoplasmic fractions was conducted for 36 hours in NTD125 expressing KB cells. NTD125 was detected by anti-HA antibodies 6 hours after transfection of NTD125-cDNA and was mostly nuclear until 33 hours after which its level dropped (Fig. 7c). Interestingly, at 6^th^ hour cytoplasmic p53 started migrating in to the nucleus (Fig. 7d). This result suggested that NTD125 stabilized cytoplasmic p53 in wild type conformation and initiated its nuclear translocation. Genotypically WT p53 in a mutant conformation promotes cell growth and behaves as a tumor suppressor only when present in the wild type conformation [27], [28]. NTD125 thus stabilized and induced nuclear translocation of cytoplasmic p53 supporting earlier observations that nuclear translocation of p53 could result in a change in the conformation from mutant to wild type [29]–[31]. In order to check whether this nuclear localized p53 is transcriptionally active, we performed luciferase reporter assay using p53 targeted gene promoter constructs such as Noxa, Bax, PUMA and p21. In NTD125- transfected cells, Noxa, Bax, PUMA and p21 promoters were shown to be activated ∼5.0, ∼7.5, ∼4.5, and ∼5.0 fold respectively (Fig. 8a). In addition, RT-PCR analysis of p21, Noxa, Bax, PUMA and SUMO genes also yielded higher RNA expression of these genes in NTD125 expressing KB cells in comparison to control cells (Fig. 8b), thus confirming that nuclear p53 was transcriptionally active.

## Discussion

Wild type p53 exists in two different conformational states, latent and active form in cells; the active form binds to DNA and is transcriptionally active whereas the latent form is devoid of these functions [32]. It exists in a conformational equilibrium between wild type and mutant conformation and equilibrium shifts in response to various stress conditions for binding to DNA and interaction with other proteins [25]. Interaction with N-ter specific antibodies PAb1801 has been shown to stabilize temperature-sensitive DNA-binding of wild-type and tumor derived mutant form of p53 through conformational stabilization [2] which suggests that NTD plays role in thermally sensitive, specific DNA binding of p53. It was proposed that p53-NTD can be involved in interdependent interaction with the C-terminus to regulate defined function of p53 [33]. CD studies of the full length p53 showed that NTD of p53 contains unstructured region in its native state [22]. Our experiments with highly purified recombinant NTD through CD analysis and IPP clearly showed that NTD125 binds to p53 and stabilized it at higher temperatures. Cryoelectron microscopy studies had earlier shown that in an intact p53 tetramer NTD of one molecule was positioned near CTD of another molecule thus forming an N/C node that was further confirmed by GST-pulldown assay and IPP [18].

We have demonstrated for the first time that the flexible p53-NTD that is devoid of tertiary structure possesses chaperone-like function in stabilizing p53 wild type conformation at higher temperature both *in vitro* and *in vivo*. Fluorescence based thermal aggregation assay, *in vitro* protection assay via IPP at various temperatures and ELISA confirmed that NTD125 displayed chaperone-like function in stabilizing the wild type conformation and restoring the mutant phenotype at elevated temperature. HSP90 and CHIP were earlier shown to stabilize WT p53 at higher temperature [24], [25].

We rationalized that the binding of NTD125 to p53-CTD might stabilize the core domain that, in turn, enhanced p53-DBS binding. Peptides were shown to stabilize p53 core domain [34] and stimulated p53-DBS binding [35]. EMSA analysis confirmed that NTD125 enhances p21-5′-DBS binding of p53 in dose dependent manner. Further, NTD125 restored p53 DBS binding of heat denatured p53 in an ATP-independent manner, unlike HSP90 [24]. Intrinsically disordered proteins were predicted to negatively correlate with the tendency of chaperone binding, although chaperone molecules binding would not assist in folding but might promote the assembly with partners in molecules [36]. p53-NTD interacts with multitude of protein factors that include molecular chaperones such as HSP90 [24], CHIP [25], HSP70 [37], and MDM2 [38] and it was shown to undergo disorder to order transition by interacting with MDM2 [39]. Short p53 TAD fragments in the intrinsically disordered p53-NTD domain were able to form ‘induced helices’ upon binding to target proteins [8], [40]. TAD2 in the NTD (aa 40–61) was also shown to fold into amphipathic alpha helices upon binding to replication protein A (RPA) [41] and Tfb1 subunit of yeast TFIIH [42].

Various synthetic compounds and small molecules have been identified that allowed mutant p53 to maintain active conformation and caused accumulation of active p53 in cells [43], [44] in order to improve antitumor therapy [45]. In KB cells that were transfected with NTD125, we have shown that p53 wild type conformation (PAb1620) was at a higher ratio than p53 mutant type conformation (PAb240) at elevated temperature. These results via IPP and *in vivo* ELISA confirmed that NTD protected and preserved WT p53 in native form. p53-NTD contains two separate transcativation domains TAD1 (aa residues 1–40), TAD2 (aa residues 40–61) and a proline-rich domain (aa residues 64–93) [1]. It is interesting that deletion of proline-rich domain (NTD55 and NTD61) resulted in loss of NTD chaperone function in cells. PRD was shown to contribute to p53 stability via Pin1 [46] and induction of p53-dependent apoptosis [47]. Earlier TAD2 along with PRD was identified for inducing pro-apoptotic genes or inhibition of anti-apoptotic genes [48]. Taken together, we concluded that transactivation domain along with PRD might be responsible for the observed chaperone-like function.

**Figure 9:**
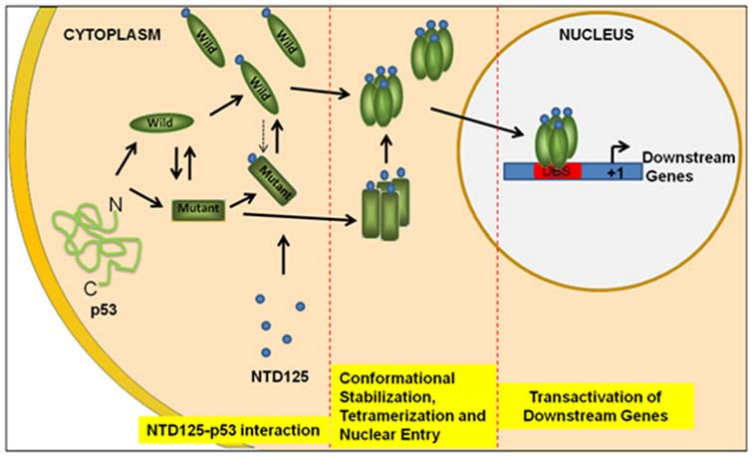
A Model for activation of p53 through NTD125- NTD125 molecules interact with cytoplasmic p53 leading to stabilization of wild conformation that further initiate nuclear translocation of activated p53. Nuclear localized p53 activates transcription of downstream genes.

Nuclear translocation of p53 can result in a change in the conformation from mutant to wild type [29], [30] and genotypically WT p53 behaves as tumor suppressor in activating p53-downstream genes [28]. In NTD125 transfected cells, post 6 hours, NTD125 was shown to co-translocate cytoplasmic p53 in to the nucleus. It is assumed that NTD125 might have triggered the activation of p53 for its nuclear translocation. The nuclear translocation of p53 can result in a change in the conformation from mutant to wild-type although these may be two separate events [29]. Under non-stress conditions, there exists equilibrium between the import and export of WT p53 in and out of nucleus. It was also proposed that p53 might be escorted to the nucleus by chaperones such as HSP90 and the binding of HSP90 to the WT p53 inhibits the formation of multiple chaperone complexes with WT p53 [37]. Recently, we have shown that molecular chaperone CHIP co-translocated WT p53 into the nucleus and activated p53 gene transcription [25]. The NTD-mediated nuclear translocation of p53 further activated p53 downstream genes such as p21, Noxa, PUMA, SUMO, Bax. Based on above observations, we propose a model of p53 activation by NTD125 that might display chaperone-like function (Fig. 9).

The chaperone-like role of NTD125 both *in vitro* and in cells raises the possibility whether WT p53 might possess a self-chaperoning role as un-cleaved molecule; intra-molecular chaperone-like fragments occur frequently in proteins and such proteins would be prone to changing conditions and in particular, to mutations in the critical building block region [49]. α-synuclein and other chaperones require co-operativity between N- and C-ter [19] and an intra-molecular interaction between N- and middle region was essential for *in vivo* function of yeast HSP90 [50]. To the best of our knowledge, this is the first report showing the presence of chaperone like activity within p53-N-ter region. As NTD125 could activate p53 signaling pathways in cells by activating its downstream genes; it would be of interest to explore the conversion of p53 mutant phenotype in to the wild type in cancer cells. Further study would aim at exploring the self chaperoning role of p53 in an intermolecular context and consequences of NTD125 expression in cellular system.

## Materials and Methods

### Plasmids, Protein purification and Antibodies

Vectors used and methods for purification of bacterially expressed His-p53, GST-p53 and GST-CHIP were described earlier [25]. pET32a-NTD125, pET21c-NTD125 and pET28a-NTD125 plasmids were used for expressing recombinant His-NTD125 in *E. coli* BL21 (DE3) cells and purified using the same protocol as His-p53. HA-tagged p53, NTD125 and deleted variants were expressed in mammalian system by cloning PCR amplified fragments in pNHA1 vector at *Xba*I/*EcoR*I site, generating, pNHA1-p53, pNHA1-NTD125, pNHA1-NTD93, pNHA1-NTD61, and pNHA1-NTD55 plasmids. Primers used for amplification are summarized in Table S1. For Luciferase reporter assay, pGL3-BAX, pGL3-Noxa, pGL3-p21, pGL3-PUMA and pSV-β-gal plasmids were used. Antibodies; anti-p53 (PAb1801), anti-p53 (PAb421), anti-p53 (PAb 1620), anti-p53 (PAb 240) were from Calbiochem); anti-p53 (C-19), anti-p53 (FL-393), anti-His, anti-GST and all secondary antibodies from Santa-Cruz and anti-HA from Babco were used.

### Cells culture, Transfection, Heat shock treatment and Immunoprecipitation

KB cells were procured from NCCS, Pune, India, and maintained in Dulbecco's modified Eagle's medium (DMEM) (Sigma-Aldrich) with 10% FCS. Transfections were carried out using either Effectene Transfection Reagent (Qiagen) or Escort™ IV Transfection Kit (Sigma) according to manufacturer's instructions. Cells were processed for sample preparation after 24 hours, 36 hours post transfection or as described in the figures. For heat shock experiment, 24 hours post-transfection, cells were grown at 42°C for 75 minute in CO_2_ incubator and then processed for IPP/*in vivo* ELISA assay. For IPP using conformation specific antibodies, cells were transfected, treated and processed as described earlier [25] except that the lysis was done in NP-40 lysis buffer (20 mM Tris-HCl pH 7.4, 100 mM NaCl, 10% Glycerol, 1.0% NP-40, 1 mM EDTA and protease inhibitor cocktail). Eluted samples were resolved on 12% SDS-PAGE and probed using PAb C-19 antibodies.

### Thermal denaturation curve and Fluorecence spectrophotometry (Aggregation assay)

The heat denaturation studies were carried out by recording the change absorbance of 100 µg of p53 or NTD125, diluted in 1 ml PBS at 280 nm wavelength in UV-visible spectrophotometer (CARY, 100 Bio, Varian) attached to temperature controller. The variation in temperature was done at the rate of 1°C/minute. The aggregation assay was performed as follows: p53 (1.0 µM) was incubated at 37°C and 45°C with or without NTD125 (2.0 or 5.0 µM). Thermal aggregation kinetics was monitored by measuring light scattering, in a fluorescence spectrophotometer (CARY Eclipse, Varian) attached to temperature controller, in the Peltier controlled thermostatted quartz cuvettes. All the measurements were done at the excitation and emission wavelengths of 340 nm with a spectral bandwidth of 5 nm.

### Circular Dichroism (CD) spectroscopy

For CD spectroscopic analysis, all the measurements were done on J-815 Circular Dichroism System (Jasco), in the Far-UV range from 260 to 195 nm using the cuvette of 0.2 cm pathlength. 100 µg of p53 or NTD125 was diluted in 1 ml 10 mM phosphate buffer (pH 7.5) and the spectra were collected at different temperatures. For each sample, an average of the three measurements was taken at a scan rate of 50 nm/minute. For interaction studies, 100 µg each of p53 and NTD125 were mixed, diluted in 1 ml 10 mM phosphate buffer (pH 7.5) and then incubated for 1 hour at the temperature at which spectra had to be recorded. For all the measurements, the spectrum of the buffer was deduced from the sample. Molar ellipticity was calculated using [molar ellipticity (θ) = millidegrees/(pathlength in millimeter × the molar concentration of proteins × the number of residues)] formula [51]. Secondary structure analysis was done using Jasco Secondary Structure Estimation ver 1.00.00 tool and CDNN software.

### *In vitro* protection and co-immunoprecipitation assay-

For *in vitro* protection assay, 2 µg of recombinant His-p53 was incubated with or without recombinant GST-CHIP (1∶2) or His-NTD125 in (1∶5) molar ratio. Protein mixture was then diluted to 100 µl in PBS (containing protease inhibitor cocktail) and incubated at different temperatures i.e. RT, 37°C, 40°C, 42°C and 45°C for 1 hour. Further, the sample was diluted to 500 µl and 1 µg of PAb C-19/PAb1620/PAb240 added to the mixtue and incubated on a rotatory shaker for 1 hour at 4°C. After that 50 µl of 10% protein-A agarose (pre-saturated with BSA) was added to the sample and incubated for 2 hours at 4°C, with continuous stirring. After pelleting, beads were washed thrice with NP-40 washing buffer (0.5% NP-40 in PBS + protease inhibitor cocktail) and finally the immunocomplex was released in 50 µl of NP-40 washing buffer by adding SDS loading dye and boiling for 3 minutes. For assessment of restoration activity of NTD125, p53 was first denatured and then NTD125 was added to the mixture and incubated at 4°C for another hour and finally immunoprecipitated using different antibodies. For co-immunoprecipitaion assay, the entire procedure was repeated as above, only His-p53 and His-NTD125 were taken in (1∶1) molar ratio. For immunoblotting 20 µl of eluted sample was resolved on 12% SDS PAGE and semi-dry blotted on to nitrocellulose membrane, western blot was developed using PAb1801.

### Electrophoretic Mobility Shift Assay

The DNA binding activity of recombinant p53 was monitored by EMSA with or without NTD125. The EMSA reaction was set using 3 ng probe and 100 ng recombinant p53, as described previously [25]. Using radio-labeled p21-5′ DBS site as probe [25], reaction mixtures were incubated for 30 minutes at RT and then loaded on to 4% native-PAGE containing 0.5 X TBE buffer, subsequently, gel was dried and exposed for autoradiography. To visualize the effect of NTD125 on the DNA binding activity of p53, recombinant NTD125 in different amount (as shown in figures) was added to the p53 and incubated for 1 hour at 37°C prior to set the reaction. Whereas to asses the restoration activity, p53 was first denatured and then incubated with NTD125 (in different molar ratios) at 4°C for 1 hour before adding to reaction mixture. We used 5 µg of isolated nuclear extract for the EMSA, when KB-nuclear extract was used as the source of WT p53.

### DNA-protein ELISA

PAb421 (0.5 µg) diluted in 50 µl PBS was coated per well and incubated at 4°C overnight, plate was then washed once with PBS and blocked using 200 µl of 1% BSA in PBS for 1 hour. Plate was then washed thrice, 5 minute each, with wash buffer (PBS + 0.05% Tween-20). After that WT p53 protein was bound to the antibody by adding 0.5 µg of p53 protein diluted in 50 µl PBS in the well and incubating it at 4°C for 1 hour. To remove the unbound protein, plate was washed thrice, 5 minute per wash, with wash buffer. Subsequently 0.5 µg of biotin labeled DBS diluted in 50 µl 1X GMS buffer was added in the well and incubated at 4°C for 1 hour. Plate was washed again three times with wash buffer, and then 50 µl of 1∶400,000 diluted Avidin Alkaline Phosphatase (AAP from Sigma) in PBS was added per well and incubated for 2 hours. Washing was again done thrice with wash buffer, and the color was developed by adding 100 µl of 1 mg/ml PNPP in AP buffer (50 mM Na_2_CO_3_, 1 mM MgCl_2_, pH 9.8) and incubated at 37°C. The reaction was then stopped by adding 100 mM EDTA, and absorbance was taken at 405 nm in the ELISA reader (Benchmark Plus Microplate reader, Bio-Rad, USA). To study the DNA binding by heat denatured p53 protein, p53 was diluted in PBS, heat denatured at 37°C for 1 hour in water bath with or without NTD125 and added on to the PAb421 coated wells, DBS was then added and bound DBS was detected using AAP.

### ELISA

An ELISA-based protein-protein interaction assay was utilized for p53- and NTD125 interaction studies. 96-well Maxisorp plates (Nunc) were coated with 50 µl of a 10 µg/ml (NTD125/CHIP/BSA) protein in PBS at 4°C over night. The wells were rinsed with cold PBS at 4°C three times. Blocking was done with 2% BSA (Sigma) in PBS at 4°C for 4 hours. Following the blocking step, the wells were washed three times with PBS containing 0.01% (v/v) Tween-20 (Sigma). p53 protein (0.5 µg) was diluted in 50 µl PBS, 0.05% (v/v) Tween-20, 0.2% (w/v) BSA and (in increasing concentration) added into NTD125/CHIP/BSA coated wells. After an incubation period of 90 minute at 4°C, the ELISA plates were washed with PBS containing 0.01% Tween-20 three times. The p53 protein was detected using 0.2 µg of mouse monoclonal antibody PAb C-19 in 50 µl PBS, 0.05% (v/v) Tween-20, 0.2% (w/v) BSA and then AP-conjugated anti-mouse secondary antibody. Finally, 100 µl of alkaline phosphate substrate i.e. 1 mg/ml PNPP in AP-buffer (pH 9.6) was added and the enzymatic reaction was allowed to take place for 30 minutes at room temperature. The reaction was terminated by adding 50 µl of 0.1 M EDTA (pH 8.0). The optical density was determined at 405 nm using Microplate reader (Bio-Rad). Total p53 concentration in cells was measured using PathScan^R^ Total p53 Sandwich Elisa Kit, from Cell Signaling Technology^R^. The endogenous p53 level was observed in mock and NTD125 transfected KB cells in a time dependent manner. Cells were collected and washed twice with PBS. The cells were lysed and processed for detection of p53 according to manufacturer's protocol. For detection of HA-NTD125, wells were coated with polyclonal p53 antibody and protein was detected using monoclonal anti-HA antibody (12CA5) conjugated with reporter enzyme HRP (Direct HA detection western blot kit, Biochemia).

### Sandwich ELISA

Investigation of the p53 conformation *in vitro* was carried out using two-site ELISA. Firstly the wells were coated with p53 conformation specific monoclonal antibody PAb1620 or PAb240 at concentration of 50 ng/100 µl per well in 0.1 M carbonate buffer (pH 9.2) at 4°C for 16 h. The wells were rinsed with PBS three times. Blocking was done with 2% BSA (Sigma) in PBS at 4°C for 2 hours. Following the blocking step, the wells were washed three times with PBS containing 0.05% (v/v) Tween-20 (Sigma). p53 protein (100 ng) was diluted in 100 µl of PBS, 0.05% (v/v) Tween-20, 0.2% (w/v) BSA with NTD125 (1∶5) or without NTD125 were incubated at different temperatures for 1 hour, added in the wells and incubated for 90 minute at 4°C. The ELISA plates were then washed with PBS containing 0.05% Tween-20 three times. p53 protein was detected using 50 ng of goat monoclonal antibody PAb C-19 in 50 µl PBS, 0.05% (v/v) Tween-20, 0.2% (w/v) BSA and then AP-conjugated anti-goat secondary antibody. Finally detection was done as described earlier. Sandwich ELISA (*in vivo* ELISA) from cell lysate was done as follows; wells were coated with 100 µl of 5 µg/ml anti-p53 antibodies (PAb1620, PAb240 and PAb C-19) overnight at 4°C. After washing thrice with TBS buffer (0.05% Tween-20 in PBS), blocking was done using 5% skimmed milk in TBS for 2 hours at 4°C. After washing the wells thrice with TBS, 200 µg cell lysate in NP-40 buffer (normal/heat shocked) 1∶1 v/v diluted in 5% skimmed milk in TBS was added to each well and incubated at 4°C for 2 hours. Subsequently 100 µl of anti-p53 polyclonal antibody Fl-393 (1∶1000 diluted) was added to each well and incubated at 4°C for 2 hours. Again after three quick washes, 100 µl of AP-conjugated anti-rabbit secondary antibody (1∶1000) was added to each well and kept at RT for another 2 hours. ELISA was developed using 100 µl of 1 mg/ml PNPP solution in AP- buffer for 30 minutes and after terminating the reaction O.D. was recorded at 405 nm on Microplate reader (Bio-Rad).

### Preparation of nuclear and cytoplasmic extracts

KB cells were scraped with a rubber policeman, and pelleted. 200 µl of cytoplasmic extraction reagent CER I (NE-PER™ Nuclear and Cytoplasmic Extraction Kit, Pierce Inc.) was added per 20 µl of packed cell volume, and the cell pellet was vortexed for 15 seconds. Cells were incubated in presence of CER I for 10 minutes, followed by incubation with 11 µl of CER II for another minute. Lysed cells were centrifuged at 13,000 rpm for 5 minutes to pellet the intact nuclei. The supernatant containing the cytoplasmic fraction was carefully separated. The pelleted nuclei were resuspended in 100 µl of NER I, votexed for 15 seconds and incubated on ice for 45 minutes with periodic vortexing after every 10 minutes. After this, the suspension was centrifuged at 13,000 rpm and the supernatants containing the nuclear proteins were stored at -80°C. Nuclear and cytoplamic fractions were checked by western blotting using anti-actin and anti-RNA pol-II antibodies (data not shown).

### Luciferase reporter assay

KB cells were plated in six-well plate the day before transfection such that they become 60–80% confluent before transfection. Reporter plasmid (1.0 µg), full length promoter-constructs were transfected together with (1.0 µg) expression vectors (pNHA1-NTD125) and (0.5 µg) β-galactosidase-expression plasmid (pSV-β-gal; Promega) per well as per the manufacturer's instructions. For each transfection, DNA content was kept uniform by using empty vector for relative plasmid type. The cells were incubated at 37°C, in CO_2_ incubator, in serum free media for 6 hours and then media was replaced with fresh complete DMEM media. After 24 hours the cells were washed in cold PBS three times and lysed with 200 µl of the 1 X lysis buffer (Promega) for 20 minutes at 4°C, the lysate was then centrifuged at 14000 rpm for 5 minute at 4°C. Supernatant was collected and 20 µl supernatant was used for the assay of luciferase activity using Luciferase reporter gene assay kit (Promega) as per the manufacturer's instruction. The β-galactosidase activity was determined using the β-galactosidase Enzyme Assay Kit (Promega). Luciferase activity was normalized by β-galactosidase activity and the data from triplicate determinations were expressed as mean ± SD.

### RNA isolation and RT-PCR

KB Cells were lysed in appropriate amount of Trizol (1 ml Trizol per well of a 6 well plate for cultured cells). Cells were repeatedly and vigorously pippetted. Cells were then kept at room temperature for 10 minutes, after which 200 µl of chloroform per 1 ml of Trizol was added and mixed thoroughly. The cells were again left at room temperature for 10 minutes. Cells were then centrifuged at 12,000 rpm at 4°C for 15 minutes and the upper aqueous colorless layer was transferred to a fresh eppendorf tube. To this eppendorf tube, 75 µl Lithium Chloride (LiCl) followed by 1 ml chilled Ethanol were added and kept at -20°C for 2-3 hours. The eppendorf tube was centrifuge at maximum speed for 15 minutes at 4°C. The supernatant was discarded, 250 µl of 70% Ethanol was added and the tube was kept at room temperature for 2 minutes. The tube was again centrifuged at 7500 rpm for 5 minutes at 4°C, the supernatant was then discarded and finally, the pellet was resuspended in RNA grade water till it was completely dissolved. Single Strand c-DNA was synthesized with sense and anti-sense primers using RevertAid ™ H Minus First Strand cDNA Synthesis Kit (Fermentas). The resulting cDNA was diluted (1∶10) before proceeding with the PCR reaction. PCR was conducted in Mastercycler gradient (Brinkmann Instruments Inc., Westbury, USA). Each 50 µl PCR reaction employed cDNA, 2.5 U Taq polymerase (Eppendorf scientific Inc., Westbury, USA), 0.2 mM dNTPs and 0.5 µM primer. PCR products were resolved on 2% agarose gel. The size of the PCR amplicon was determined by comparison with 100-bp DNA ladder (Promega, Madison, USA). Primers used for amplification of appropriate gene are summarized in Table S2.

## Supporting Information

Table S1 Primers for PCR amplification of p53, NTD125 and NTD-variants; for cloning in pNHA1 plasmid vector.
(0.06 MB RTF)

Table S2 Primers for RT-PCR analysis.
(0.06 MB RTF)
