# Supplementary material for: p53 Amino-Terminus Region (1–125) Stabilizes and Restores Heat Denatured p53 Wild Phenotype
Source: PLoS One. 2009 Oct 22;4(10):e7159. doi: 10.1371/journal.pone.0007159 (PMC2760748; doi:10.1371/journal.pone.0007159)
Supplement: Table S1 — Primers for PCR amplification of p53, NTD125 and NTD-variants; for cloning in pNHA1 plasmid vector. (0.06 MB RTF) [file pone.0007159.s001.rtf]

Table. S1
Table. S1. Primers for PCR amplification of p53, NTD and NTD-varients; for cloning in pNHA1 plasmid vector. 	
	For All 	
sense primer 	5'-CTAGCTAGCTCTAGAATGGAGGAGCCCCAGTCAGATCC-3' 	
	For p53 	
anti-sense 	5'-CCGGAATTCTAGCTCTAGAGTCTGAGTCAGGCCCTTC-3' 	
	For NTD-125 	
anti-sense 	5'-CCGGAATTCTTACGTGCAAGTCACAGACTTGGC-3' 	
	For NTD-93 	
anti-sense 	5'-CCGGAATTCTTACAGGGGCCAGGAGGGGGCTGG-3' 	
	For NTD-61 	
anti-sense 	5'-CCGGAATTCTTAATCTGGACCTGGGTCTTCAGTGAACC-3' 	
	For NTD-55 	
anti-sense 	5'-CCGGAATTCTTATTGGGACGGCAAGGGGGACAG-3' 	
