# Supplementary material for: p53 Amino-Terminus Region (1–125) Stabilizes and Restores Heat Denatured p53 Wild Phenotype
Source: PLoS One. 2009 Oct 22;4(10):e7159. doi: 10.1371/journal.pone.0007159 (PMC2760748; doi:10.1371/journal.pone.0007159)
Supplement: Table S2 — Primers for RT-PCR analysis. (0.06 MB RTF) [file pone.0007159.s002.rtf]

Table. S2. Primers for RT-PCR analysis 	
	For p21WAF1/CIP1 	
sense primer	5'-ATGAAATTCACCCCCTTTCC-3'	
antisense primer	5'CCCTAGGCTGTGCTCACTTC-3'	
	For BAX 	
sense primer	5'-AGAGGATGATTGCCGCCGT-3'	
antisense primer	5'-CAACCACCCTGGTCTTGGAT-3'	
	For NOXA 	
sense primer	5'-CTGGAAGTCGAGTGTGCTACT-3'	
antisense primer	5'-TCAGGTTCCTGAGCAGAAGAG-3'	
	For SUMO 	
sense primer	5'-ACCGTCATCATGTCTGACCA-3'	
antisense primer	5'-TGGAACACCCTGTCTTTGAC-3'	
	For PUMA 	
sense primer	5'-CTGTGAATCCTGTGCTCTGC-3'	
antisense primer	5'-TCCTCCCTCTTCCGAGATTT-3'	
	For â-Actin 	
sense primer	5'-CGGTTGGCCTTAGGGTTCAGGGGGG-3'	
antisense primer	5'-GTGGGCCGCTCTACGCACCA-3'	
Table. S2
